# Freshwater Cyanobacterial Toxins, Cyanopeptides and Neurodegenerative Diseases

**DOI:** 10.3390/toxins15030233

**Published:** 2023-03-21

**Authors:** Galina Nugumanova, Eugene D. Ponomarev, Sholpan Askarova, Elizaveta Fasler-Kan, Natasha S. Barteneva

**Affiliations:** 1 Department of Biology, School of Sciences and Humanities, Nazarbayev University, Astana 010000, Kazakhstan; 2 Center for Life Sciences, National Laboratory Astana, Nazarbayev University, Astana 010000, Kazakhstan; 3 Department of Pediatric Surgery, Children’s Hospital, Inselspital Bern, University of Bern, 3010 Bern, Switzerland; 4 The Environment & Resource Efficiency Cluster (EREC), Nazarbayev University, Astana 010000, Kazakhstan

**Keywords:** cyanotoxins, cyanopeptides, harmful algal blooms, neurodegenerative disease, microcystin, BMAA, non-proteogenic amino acids, mistranslation, amyotrophic lateral sclerosis, Parkinson’s disease, Alzheimer’s disease, gut-brain axis

## Abstract

Cyanobacteria produce a wide range of structurally diverse cyanotoxins and bioactive cyanopeptides in freshwater, marine, and terrestrial ecosystems. The health significance of these metabolites, which include genotoxic- and neurotoxic agents, is confirmed by continued associations between the occurrence of animal and human acute toxic events and, in the long term, by associations between cyanobacteria and neurodegenerative diseases. Major mechanisms related to the neurotoxicity of cyanobacteria compounds include (1) blocking of key proteins and channels; (2) inhibition of essential enzymes in mammalian cells such as protein phosphatases and phosphoprotein phosphatases as well as new molecular targets such as toll-like receptors 4 and 8. One of the widely discussed implicated mechanisms includes a misincorporation of cyanobacterial non-proteogenic amino acids. Recent research provides evidence that non-proteinogenic amino acid BMAA produced by cyanobacteria have multiple effects on translation process and bypasses the proof-reading ability of the aminoacyl-tRNA-synthetase. Aberrant proteins generated by non-canonical translation may be a factor in neuronal death and neurodegeneration. We hypothesize that the production of cyanopeptides and non-canonical amino acids is a more general mechanism, leading to mistranslation, affecting protein homeostasis, and targeting mitochondria in eukaryotic cells. It can be evolutionarily ancient and initially developed to control phytoplankton communities during algal blooms. Outcompeting gut symbiotic microorganisms may lead to dysbiosis, increased gut permeability, a shift in blood-brain-barrier functionality, and eventually, mitochondrial dysfunction in high-energy demanding neurons. A better understanding of the interaction between cyanopeptides metabolism and the nervous system will be crucial to target or to prevent neurodegenerative diseases.

## 1. Introduction

Cyanobacteria are a group of photosynthetic microbes inhabiting diverse aquatic freshwater, marine, and terrestrial environments. Cyanotoxin production is considered to be an ancient trade exceeding 2.5 billion years of age [1].

The ability of cyanobacteria to produce cyanotoxins and their ubiquity in freshwater ecosystems with increasing demands upon water resources require better detection and a more comprehensive understanding of cyanobacterial distribution and its impact on animals and humans. Although cyanobacterial cell numbers change seasonally, the toxins can persist in water for several months, extending low-dose exposure [2]. Aquatic organisms (e.g., grazers/herbivores, fish, mammals) may consume aquatic plants with high concentrations of cyanotoxins, and trophic transfer may happen to higher-order organisms, although, for some toxins, biodilution, and not biomagnification may be a predominant process in the food webs [3]. The detectable presence of cyanobacterial toxins in animal tissues has been found to be associated with mass mortalities of animals, including cows, dogs, and sea mammals [4,5,6,7,8,9,10,11,12], birds [13], and some human cases [14,15]. The terrestrial vertebrates affected by cyanotoxins are more diverse than was thought before [16,17]. Moreover, cyanotoxins demonstrate sublethal effects, including growth inhibition in zooplankton and aquatic plants, macroinvertebrates, and aquatic plants [18].

The predicted climate changes, such as increased water temperatures, together with anthropogenic nutrient loadings, escalate freshwater cyanobacterial frequency, duration, and size of algal blooms [19,20,21]. The cyanobacterial abundance has increased disproportionally relative to other phytoplankton since 1945 [22]. Although our knowledge about cyanotoxins mostly comes from temperate and tropic areas, Arctic regions undergo the most pronounced and rapid climate changes, and high-latitude lakes support cyanobacteria blooming and cyanotoxins production [23]. Moreover, the occurrence and intensity of near-surface phytoplankton harmful algal blooms (HABs) have been increasing across the world [24,25] due to the eutrophication or nutrient enrichment of water bodies [26,27]. The potential presence of low doses of cyanobacterial toxins in drinking water is likely to be a continuing problem. 

## 2. Cyanobacterial Toxins

Cyanobacteria produce a variety of toxins. Traditionally, they are divided based on functional properties into main groups: hepatotoxins, neurotoxins, dermatotoxins, and cytotoxins. The toxins found in cyanoHABs include cyclic peptides microcystins (MCs), and nodularins, and neurotoxins such as anatoxins (anatoxin-a, homoanatoxin-a, guanitoxin), ciguatoxins, saxitoxins, ß-methylamino-L-alanine (BMAA) and its isomers (2,4-diaminobutyric acid (2,4-DAB) and N-(2-aminoethyl)-glycine (AEG) [21]. More than 80 cyanobacteria species are known to be toxigenic, and assays for the detection and toxicity of cyanotoxins continue to develop. 

One of the major sources of cyanotoxins for humans is drinking water. However, guidelines for water quality monitoring are limited to MCs [28]. Furthermore, exposure of grazing livestock with terrestrial cyanobacteria that may be present in fields used for livestock grazing (biofilms of *Phormidium* sp.) or can be fed to livestock directly [29,30,31,32], and of which the aggregates can bioaccumulate in cow’s milk [29,33] or bird eggs [34] and which increases the likelihood of human exposure [35]. There is an urgent need to detect other cyanobacterial toxins in drinking water and food and to understand how they are involved in the pathogenesis of chronic diseases such as neurodegenerative disorders. 

Many cyanobacterial toxins still have to be discovered. For major toxins groups, new variants can be found and characterized. Thus, a structural variant of anatoxin-a, dihydro-anatoxin-a, has been recently identified in many samples of benthic cyanobacteria, even exceeding the abundance of anatoxin-a [36]. Of potential interest are compounds with neurotoxic effects, such as cyanostatins A and B, lipopeptides, isolated from cyanobacterial water blooms [37], anabaenopeptins [38], and microginins [39]. The identification of biologically active cyanotoxins, including many novel lipopeptides with neurotoxic effects such as jamaicamides and antillatoxins continues [40]. Vacuolar spongiform myelopathy has recently been linked to aetokthonotoxin (AETX) from epiphytic cyanobacterium *Aetokhonos hydrillicola* that is growing in man-made water bodies of the southeastern United States [41]. This finding warrants further research into the potential toxins produced by epiphytic and benthic cyanobacteria species. 

### 2.1. Microcystins (MC) Family 

A full structural chemical analysis of MCs was achieved in the 1980s through a combination of spectroscopy, nuclear magnetic resonance, mass spectrometry, and amino acid analysis and demonstrated that the chemical structure of MCs consists of a cyclic heptapeptide with two variable and five relatively conserved amino acids biosynthesized non-ribosomally via an MC synthetase gene cluster [42]. A universal nomenclature system was suggested based on the positions of amino acid residues 2 and 4 (denoted as X and Z in the original structure of MC, i.e., MC-XZ) [43]. By 2019 the identification of at least 279 different MC congeners was reported in the literature [42].

Among cyanobacterial toxins, MCs are the most diverse group and the best described, though MC-LR and MC-RR variants—are the only two widely researched. Other MC congeners demonstrate different toxicokinetic and toxicodynamic features [44]. Variations in toxicity between MC congeners can be attributed to the differences in their uptake by organic anion transporting polypeptides (OATP) transport as well as changes in their inhibition of serine/threonine protein phosphatases (PP) 1 and 2a [45]. The toxicity of MCs depends on variations in their chemical structure and ranges over six orders of magnitude [46]. 

MC toxicity affects not only the liver but also the brain [47] and other organs. Multiple neurotoxic effects of MC-LR were demonstrated using multiple biological models, including birds, fishes, and mammals [48,49,50]. For example, using murine brain cell line as a model, congener-dependent pronounced neurodegenerative effects were identified (MC-LF >> MC-LW > MC-LR) [51]. 

### 2.2. BMAA (β-N-methylamino-l-alanine) and Isomers

BMAA is a non-proteogenic amino acid produced by all known groups of free-living and symbiotic cyanobacteria [52]. The isomeric forms of BMAA, such as 2,4-DAB and AEG, can also be found in different species of cyanobacteria, including *Anabaena*, *Leptolyngbya* sp., *Oscillatoria* sp., *Merismopedia* sp. and *Microcystis aeruginosa* [53]. The isomers are detected in nature along with BMAA but are less studied. BMAA isomers are neurotoxic [54]. Recent research using larval zebrafish as a biological model identified 2,4-DAB as a more potent neurotoxin than AEG and BMAA [55].

Despite some contradictory research [56], an increasing body of experimental proof provides confirmation that BMAA plays a significant role in neurodegenerative diseases (ND) [57,58,59]. Some analytical methods related to liquid chromatography and fluorescence-based detection of BMAA, especially in older literature, may be prone to overestimation of BMAA concentration in studied samples and should be treated cautiously [60,61]. BMAA has been shown to contribute to protein misfolding, enzyme inhibition, and neuroinflammation [62]. BMAA toxic effects were found to be associated with the misincorporation of serine in multiple human proteins [63,64], which can lead to the formation of inclusion bodies in neurons [65]. Misincorporation in some serine sites has been reported to contribute to neuropathologies [66]. Even a low misincorporation rate (1 per 10,000 codons) can lead to neurodegeneration in a rodent model [67]. In their in vitro and in vivo studies, several groups that BMAA leads to the overexpression TDP-43 (TAR DNA-binding protein 43) encoded by the TARDBP gene [68,69,70].

Most animal models used for studying BMAA were based on investigating BMAA effects in the brain and other organs [59]. Thus, Xie and co-authors reported that in their experiments, less than 1% of total BMAA detected in adult mice plasma was taken in the brain [71], i.e., blood-brain barrier (BBB) was not easily permeable for BMAA. The mechanism of neurotoxicity may involve a direct action on the N-methyl-D-aspartate (NMDA) receptor, activation of glutamate receptor 5, and induction of oxidative stress.

Recently, Han and co-authors [72] found that BMAA can serve as a substrate for human alanyl-tRNA synthetase (AlaRS), avoiding the intrinsic editing activity of AlaRS, acting as a competitive inhibitor, and compromising the editing ability of AlaRS. Terminally differentiated cells, such as neurons, are particularly susceptible to mistranslation and accumulation and forming of misfolded and aggregated proteins [67,73].

### 2.3. Other Cyanobacterial Neurotoxins

Traditional neurotoxins from cyanobacteria with acute effects include alkaloid or organophosphorus compounds such as (a) anatoxin-a and homologs, which affect nicotinic acetylcholine alkaloid toxins and muscarinic acetylcholine receptors [74,75]; (b) saxitoxins can be produced by both dinoflagellates and by cyanobacteria from several genera including *Aphanizomenon*, *Cylindrospermopsis*, and *Dolichospermum*; (c) guanitoxins which are similar in structure to organophosphates and able to irreversibly inhibit acetylcholinesterase [76]; their presence was also found in desert assemblages [77,78]. Cylindrospermopsin is another frequent finding during fish kills resulting from cyanoHABs [79,80]. It is an alkaloid consisting of a tricyclic guanidine moiety combined with hydroxymethyluracil [81], which interferes with cellular metabolism and causes hepatotoxic and genotoxic effects, as well as neurotoxic effects [82]. 

Nodularins can be found and bioaccumulated in a wide range of organisms [83]. Acetylcholinesterase activity (AChE) is an indicator of neurotoxic effects, and exposure to a cyanobacterial pentopeptide nodularin affects the AChE activity in the Baltic clam *Macoma balthica* [84] and the mussel *Mytilus edulis* [85].

It is known that cyanobacteria are capable of producing different toxins which can be present during the same HABs [86]. However, it is not clear, how the environmental factors regulate the abundance of different MC congeners and isoforms of other toxins in a bloom [42]. During harmful blooms of cyanobacteria, different cyanobacteria species can co-occur, potentially producing various cyanotoxins, cyanopeptides, and other metabolites simultaneously. Thus, BMAA can co-occur with its isomers (2-DAB and AEG) and have synergistic neurotoxic effects, as demonstrated in in vitro cell line experiments [87]. Moreover, the joint presence of MC-LR and BMAA leads to their interaction in vivo and to the neurotoxic effect enhancement [88]. The development of methods allowing for the assessment of multiple toxins during algal blooms is needed [89].

### 2.4. Cyanopeptides

The majority of cyanobacterial secondary metabolites are peptides or include peptidic substructures. Cyanopeptides are non-ribosomal peptides rich with posttranslational modifications and non-proteinogenic amino acids and consist of linear, cyclic, or multicyclic molecules with basic, depsipeptidic, or lipopeptidic structures. More than 500 cyanopeptides ranging from app. 300 to 2000 Da had been structurally identified by 2019 [90]. Natural selection did not minimize the pool of peptides but favors the production of a wide array of different peptide structures. The biosynthetic non-ribosomal pathways of peptide synthesis are evolutionarily ancient and precede synthetic pathways of higher plants and animals. During HABs, cyanobacteria produce a tremendous amount of diverse cyanopeptides; however, their ecological significance is unclear. They can happen at surface waters in the same nanomolar concentration as MCs, exhibit toxicity towards grazers in the same micromolar range as MCs, and their production is synchronized with *Microcystis* sp. Although the abundance of MCs can be monitored successfully, more studies on cyanopeptides appearance and persistence during blooms [90,91] and their potential for chronic toxicity are needed. The bioactive compounds produced by cyanobacteria are not limited by peptides and also include alkaloids, cyclophanes, terpenes, lactones, etc. Cyanobacterial compounds have a broad bioactive spectrum, with many acting as serine protease inhibitors, trypsin and chymotrypsin inhibitors, and anti-cancer compounds capable of modulating infectious diseases [92]. 

### 2.5. Chronic Effects of Cyanobacterial Toxins

The epidemiological studies of human health impacts of chronic cyanobacterial toxins exposure are nascent. They have been associated with neurodegenerative diseases (ND), including Alzheimer’s disease (AD), Parkinson’s disease (PD), and amyotrophic lateral sclerosis (ALS) [93,94,95,96,97]. Clusters of ALS and ALS-like diseases have been reported in relation to cyanobacteria in Guam, France, Japan, New Hampshire, and Wisconsin (summarized in Table 1). The slow onset of ND (time distance between exposure and possible outcome) and problems with the assessment of environmental exposure interfere with our understanding of the role and significance of cyanotoxins in ND.

The link between BMAA and ND is yet to be further elucidated. Several studies have reported the presence of BMAA post-mortem in the brain tissues of patients who die from ALS/PD [102,121]. However, in ALS/AD disease [122], and other ND studies some researchers were not able to identify BMAA presence [123,124]. The ALS/PD neurodegenerative disorder, formerly hyperendemic in Guam-USA, Kii-Japan, and Papua-Indonesia associated with several cycad food toxins, including cyanotoxins [125]. BMAA and its isomers have now been identified in both aquatic and terrestrial eco-systems in North America [95,96], The Baltic Sea [126], France [113], Sweden [127], Peru [128], and Qatar [129]; and is produced by several different cyanobacteria [52], diatoms [127], and dinoflagellates [130].

Residential exposure to environmental pollutants may play an essential role in the etiology of ALS, which is supported by non-random distribution by addresses of ALS patients [131].

### 2.6. Stability of Cyanotoxins

Cyanotoxins possessing cyclic peptide structures, such as MCs and nodularins, are resistant to chemical degradation [132,133], highly stable, and may persist in aquatic ecosystems for weeks and months [2,133]. Thus, MCs can be retained in mussels (*Mytilus californians*) for up to eight weeks [134]. The high stability of MCs, cylindrospermopsin [135,136], and other cyanotoxins over a wide range of pH and temperature might have significant consequences for aquatic ecosystems and contribute to bioaccumulation of toxins to higher levels of food chains. These peptides are synthesized non-ribosomally and may contain non-proteinogenic amino acids [137,138,139].

Toxins undergoing attenuation via photodegradation may vary depending on the type of toxin, HABs timing, and environmental conditions [140]. Though cyanotoxins are resistant to chemical degradation, recent advances in molecular microbial communities research have found that toxic cyanoHABs favor the specific members of bacterioplankton with degrading abilities towards cyanotoxins [141,142]. More than 120 taxa of viruses, bacteria, microfungi, heterotrophic protists, and several eukaryotic microalgae negatively affect *Microcystis* growth [143]. The strains of the bacterial genera *Sphingomonas* (majority of MC-degrading bacteria), *Rhodococcus*, *Brevibacterium*, *Burkholderia*, *Mycobacterium*, *Pseudomonas*, *Novosphyngobium* and others can degrade MCs in time scale from hours to days [144,145,146,147,148,149]. The genomes of some MCs-degrading bacteria are sequenced [150,151], and *mlr* gene cluster have been implicated in playing a prominent role in the sequential hydrolysis of MCs peptide bonds [151,152].

Similar to MCs, nodularin and cylindrospermopsin can also be degraded by bacteria isolated from cyanobacterial blooms (*Bacillus* sp., *Aeromonas* sp.) [153,154,155]. Not only bacteria but fungi demonstrate algicidal activities such as *Trichoderma citrinoviride* degrading MCs [156]. Furthermore, Mohamed and co-authors [157] summarized data on six fungal species with biodegrading activities against MCs. Between zooplankton grazers, metazoans, such as *Daphnia* and copepods, are also susceptible to cyanotoxins [158,159]. Protozoa, on the other hand, are highly resistant to cyanotoxins and demonstrate great potential in controlling harmful cyanobacteria and improving phytoplankton composition in eutrophic waters [160,161].

### 2.7. Current Cyanotoxins Analytical Methods

Analytical techniques (fraction analysis, quality control) play a critical role in assessing the cyanotoxins’ effects and were summarized in many excellent reviews [162,163,164,165,166]. There are still significant data gaps in analytical methods, including (a) the absence of all the relevant standards [167]; (b) the need for validated methodologies for cyanotoxins outside the water samples: (c) the need for standardization of cyanotoxins in multicenter monitoring programs [168]; (d) the need for new technologies allowing simultaneous identification of as many toxins as possible; (e) and the need to improve robustness and a detection limit of detection methods. Another critical challenge is analyze cyanotoxins faster and feasibly in situ [169]. Unsuitable analytical methods may partly explain the lack of consensus over the widespread presence of some cyanotoxins (BMAA) in aquatic ecosystems [170].

Recently, high-resolution mass spectrometry (HRMS/MS) has become more available for researchers. Current methods rely on defined cyanotoxins and cyanopeptides targets and are generally inappropriate for detecting and identifying emerging novel compounds. The recent approach of the non-targeted analysis of pollutants and toxins in water focus on a comprehensive workflow for the acquisition and treatment of the data generated after liquid chromatography coupled with high-resolution mass spectrometry (LC-HRMS) analysis [171,172,173,174]. So-called suspect screening identifies novel compounds, including cyanotoxins and cyanopeptides based on the exact mass (*m/z*), presence of one or more charge states (z = 1, 2, etc.), expected isotope pattern and common adducts) as well as secondary fragmentation (MS2) even without reference chemicals [175,176,177,178].

Since the middle of the twentieth century, when the first MCs were identified and purified [179], many methods have been developed to analyze environmental samples for cyanotoxins [180]. Nowadays, the number of studied cyanotoxins and analytical methods for their qualitative and quantitative detections have increased. Detection approaches vary in terms of accuracy, sensitivity, and specificity. The most commonly used techniques for cyanotoxins detection are enzyme-linked immunosorbent assays (ELISA), protein phosphatase inhibition assay (PPIA), molecular assays - polymerase chain reaction (PCR), and quantitative real-time PCR (qPCR) for toxins producing genotypes for cyanobacteria identification, liquid chromatography (LC) and high-performance liquid chromatography (HPLC) combined with different detectors. Among these, liquid chromatography-mass spectrometry (LC-MS) takes a special place because it identifies the target cyanotoxins with high accuracy at a significantly low detection level (Table 2) [181,182]. Moreover, despite the structural diversity of cyanotoxins, LC-MS allows for determining groups of toxins simultaneously. That is one extra advantage of using this detection technique integrated with bioassays and molecular assays in complex environmental samples for complete water quality assessment [183,184].

As separation instruments, HPLC and UHPLC are usually used. UHPLC is faster due to the higher pressure applied, and the online SPE procedure provides reduced sample time processing [2,199]. HPLC-UV/PDA [191], and HPLC-DAD [204] have less LOD values (3–4 μg/L, 0.2–0.3 μg/L, respectively) for MCs than LC-MS/MS where minimum LOD values vary within 0.0003–0.1 µg/L [182,196,206]. Concerning other cyanotoxins, MS detection techniques also provide relatively low values of LOD and LOQ. The LC-MS method requires expensive instruments, lengthy operator training, and thorough sample preparation, making it time-consuming. That limits LC-MS techniques’ application as ubiquitous [2]. Nevertheless, this method remains preferable for precise quantitative analysis of cyanotoxins in water samples. 

## 3. Toxin Exposure Pathways

Major cyanotoxins’ exposure routes include ingestion through drinking water or dermal contact with recreational waters [214,215], also through food, and inhalation since cyanotoxins were identified in aerosols generated by HABs [216]. Historically, cyanoHABs were considered a public health threat to freshwater lakes, rivers, and reservoirs. However, freshwater-sourced MCs can accumulate in marine mollusks in concentrations 100-fold greater than in surrounding water [10,134]. 

### 3.1. Transport of Cyanotoxins in Freshwater and Marine Systems

Recent studies demonstrated that cyanotoxins could persist during transport into estuarine and marine waters and directly affect marine ecosystems [10,134,217,218,219]. MCs and other toxins produced by freshwater cyanobacteria can enter the marine ecosystem via freshwater channels and outflows [10,218]. It changes the HABs’ management approach, requiring monitoring of multiple toxins across the freshwater-to-marine continuum and including cyanotoxins in marine and estuarine monitoring [220]. 

### 3.2. Toxin Exposure Pathways: Oral (Drinking Water)

When drinking water is impacted by cyanobacterial toxins resulting from HABs and not treated adequately to reduce the cyanotoxin levels, it can cause severe effects on the entire region [216]. 

The causes of cyanobacteria proliferation in urban environments are mainly due to the disposal of untreated domestic sewage in water reservoirs and surface runoff water from soils. In analyzing sewage disposal systems in the main cities of Kazakhstan-Almaty and Astana, the efficiency of biogenic compounds removal remain unsatisfactory, reaching only 30–40%. This eutrophication is due to the increase in nutrients, such as phosphorus and nitrogen, arising from human action, representing a serious risk to the health of living beings and drastically reducing water quality. To cope with this problem, the possibility of intensifying nitrogen and phosphorus removal using zeolite as a biofilm carrier in an activated sludge tank is examined [221]. 

### 3.3. Toxin Exposure Pathways: Oral (Food)

Food is an important source of cyanotoxin exposure [222,223] BMAA accumulation in shellfish and fish [130,223,224,225], and further transmission along the food chain (chicken tissues) [226] may lead to human exposure. Worryingly, exposure of crop plants to cyanotoxins through irrigation was already demonstrated [227,228,229]. For centuries some species of *Nostoc*—the symbiotic colonial cyanobacteria *N. flagelliforme*, *N. commune*, and *N. sphaeroides*—have been wild-harvested and consumed as a part of the traditional diet by indigenous people in different countries, including Peru, China, Ecuador, Mexico, Fiji, Philippines, Mongolia [230,231,232,233]. Cyanotoxins BMAA and its isomers were detected in dietary supplements [234,235,236].

Chronic dietary exposure to BMAA present in the traditional Chamorro diet was associated with the formation of both β-amyloid deposits and neurofibrils tangles (NFT) found in brain tissues of Chamorros people who died with ALS/Parkinson’s dementia complex (ALS/PD) [65]. BMAA occurs not only as a free amino acid at different levels of the trophic chain (cyanobacteria *Nostoc* sp., root symbioses, cycad seeds, flying foxes, and brain tissues of Chamorro people who passed away from ALS/PD) but can also be released by acid hydrolysis increasing in concentrations 10- to 240-fold [237]. Vervet monkeys fed for only six months with BMAA-dosed fruit developed β-amyloid deposits and NFT in the brain. Increasing the amount of L-serine in the vervets diet reduced the density of NFT and the risk of neurodegenerative pathological brain findings [65]. 

### 3.4. Toxin Exposure Pathways: Air (Aerosolization)

Algae can be dispersed by air [238], and aerosol can be created from the algae during HABs [239]. The increase in the salinity of freshwater streams is likely to influence the abundance and diversity of aerosolized bacteria [240]. The cyanotoxins may be transported in aerosols from lakes with high concentrations of toxigenic cyanobacteria [238,241,242,243,244,245]. Airborne cyanobacteria persist in an urban environment and indoor living quarters [246,247,248,249]. The presence of airborne cyanobacteria in the nearby beach area is significant, and representatives of different taxa, including toxic microalgae, have been studied by many researchers worldwide [245,250]. The screening for toxins in extreme habitats demonstrated their presence in all general extremophile habitats [251].

Recent findings with rat models confirmed that BMAA exposure was insufficient in producing gross toxic effects; however, it still leaves the possibility of lifelong exposure via inhalation [252]. The health concerns associated with aerosolization remain understudied [253]. 

MC-LR exposure in the existing rodent models increases lung infiltration with granulocytes [254,255] and increases proinflammatory cytokine expression [256,257]. Recently, Breidenbach and co-authors [258] reported that the human airway epithelium response to MC-LR is represented by proinflammatory phenotype, including chemokines. 

The aerosolization of cyanobacteria was proposed as a risk factor for ALS [112]. Aerial link of exposure was investigated with ALS/PD. BMAA and its isomers (DAB and AEG) were measured in air filters around lake Mascoma [96]. Moreover, Facciponte and co-authors [259] found that humans routinely inhale aerosolized cyanobacteria. Using PCR, authors identified cyanobacteria at high frequencies in the upper respiratory tract (93.20%) and central airway (79.31%). They concluded that cyanobacteria exposure might be a prevalent and chronic phenomenon and not necessarily restricted to water bodies.

Autoradiographic imaging in mice showed a distinct localization of radioactivity in olfactory mucosa and bulb following intranasal instillation of radiolabelled BMAA, confirming a direct transfer of BMAA via olfactory pathways to mice brain circumventing the BBB [260]. 

### 3.5. Natural Model of Toxin Exposure

The complexity of ND requires a deep understanding of the disease biology and makes it challenging to develop a model of cyanotoxin exposure close to neurodegenerative findings in humans due to the species-specific variations in the phosphorylation and cleavage of the tau protein [261]. Natural animal models should recapitulate two major features of human ND: Aβ deposition and NFT formation. Chronic low BMAA concentrations induce neurodegenerative changes in non-human primates [65,262]. BMAA can bioaccumulate in marine apex predators such as dolphins and sharks, and in humans [230,263,264]. It was detected in the brains of stranded dolphins with pathological hallmarks of AD at concentrations higher than those found post-mortem in individuals with ALS and AD [265]. Chronic low BMAA concentrations induce neurodegenerative changes in non-human primates [65,262]. There are increased numbers of β-amyloid+ and dystrophic neurites in the auditory cortex compared to the visual cortex and brainstem [265].

### 3.6. Cyanotoxins and Infections

BMAA can facilitate most of the mechanisms related to neurodegeneration [61]. Thus, Lobner and co-authors [266] demonstrated that BMAA at the 10–100 µmol potentiates neurotoxicity induced by β-amyloid and NMDA.

STX doubled the quantity of ZIKV-induced neural cell death in progenitor areas of human brain organoids, and the chronic ingestion of water contaminated with STX before and during gestation caused brain abnormalities in offspring of ZIKV-infected immunocompetent C57BL/6J mice. These results raised a public health concern regarding the consequences of arbovirus outbreaks in areas with droughts and/or frequent freshwater cyanobacterial blooms [267].

The outbreak of Zika syndrome coincided with a major drought in the region between 2012 and 2016. Characteristic of dry seasons, the concentration of nutrients from untreated effluents and lower volume of water, and an increase in atmospheric temperature allowed greater blooming of cyanobacteria. Consequently, the concentration of cyanotoxins, such as saxitoxins, increased. It led authors to formulate the hypothesis that cyanobacteria in the water supply would be a causal cofactor of zika-associated microcephaly. 

## 4. Mechanisms of Brain Toxicity

The toxins produced by cyanobacteria are incredibly diverse. Well-studied neurotoxins of algal origin are alkaloids saxitoxins (STXs) that have been identified in dinoflagellates and several cyanobacterial genera, including *Anabaena, Aphanizomenon*, *Planktothrix*, *Cylindrospermopsis*, and *Scytonema* [268,269]. STXs are represented by more than 50 structural analogs commonly known as paralytic shellfish toxins (PSTs) [75,268,269]. They block the passage of sodium across a biological membrane and interfere with potassium and calcium-mediated ion channels [270].

Although the pathophysiology of some alkaloid and phosphororganic toxins (STXs, anatoxins, etc.) are relatively well studied, others, such as ciguatera-like toxins, are not clear [271,272]. Recently, the neurotoxic effects of cyanopeptides attracted more attention [90,273,274]. Some cyanopeptides have been found to have anti-proliferative effects on tubulin and microtubules [273,274,275,276,277], which are crucial components for neurons. For instance, the toxic cyclodepsipeptides known as cryptophycins [273], which are 100–1000 times more potent than paclitaxel and vinblastine, can impede the formation of a proper mitotic spindle by preventing the correct assembly of microtubules. This can lead to cell cycle arrest and, ultimately, cell death [275,276,277]. Another cyclic depsipeptide apratoxin isolated from *Lyngbya* sp. induces G1 cell cycle arrest [276,277]. Major mechanisms related to the neurotoxicity of cyanobacteria compounds include (1) blocking of essential proteins and channels; (2) inhibition of essential enzymes in mammalian cells such as protein phosphatases and phosphoprotein phosphatases [45,278,279]; (3) potentially molecular targets may include toll-like receptors (TLR) 4 and 8 [280], participants in neuronal conduction and neuroinflammation [281]. The hypothesis that L-BMAA can be misincorporated into proteins is discussed in detail by Dunlop and co-authors [63]. 

In the high-profile case of fatal human intoxication with MC in the hemodialysis unit in Brazil (1996), patients developed symptoms of acute neurointoxication (intermittent blindness, deafness. convulsions, tinnitus) and subsequential hepatotoxicity [14]. The mechanisms of MCs neurotoxicity were reviewed by different researchers [282,283]. Thus, MC-LR can induce apoptosis and atrophy of gonadotropin-releasing hormone (GnRH) neurons in rats’ hypothalamus [284,285]. Although the molecular mechanism of MC-LR- induced apoptosis remains elusive, growing evidence supports that MCs and other cyanobacterial toxins, such as cylindrospermopsin, act as endocrine disruptors [286,287]. Recently, Shi and co-authors [288] demonstrated that acutely administrated MC-LR induces pathological changes in rat’s hypothalamus and pituitary gland and alters the transcription of genes involved in hormone biosynthesis. 

In recent decades an increasing number of studies showed MC-induced immune dysfunction and a disturbance in the production cytokines leading to inflammation in fishes [289,290] and rodents [291,292,293]. BMAA is proven to be neurotoxic for different biological models, including insects [294,295] and vertebrates (fish, rodents, dolphins, and birds) [40,296]. 

### 4.1. Neurodevelopmental Effects

The link between neurodegeneration and neonatal BMAA exposure, dose-dependent neuronal loss, beta-amyloid deposition, and behavioral deficits was recently demonstrated in a rat model [297]. Autoradiographic imaging confirmed transplacental uptake of radiolabelled BMAA and specific uptake in mouse fetuses [298]. Furthermore, in neonatal rats, the free BMAA concentration was higher in the neonatal brain than in peripheral tissues such as the thymus, pancreas, and spleen, except for the liver. The level of protein-associated BMAA was significantly higher in the hippocampus than in other brain regions [299]. The BMAA exposure to neural stem cells decreased neurite outgrowth and a number of neurites in neural stem cells (NSC) [300], and NSC were more sensitive to BMAA exposure than primary neurons [301]. The authors conclude that BMAA acts as a developmental toxin. BMAA can negatively impact NSC homeostasis, increasing susceptibility to neurodegenerative disease later in life [300]. Perinatal exposure in mice, even with low doses of BMAA, leads to neurobehavioral disturbances during the postnatal period and adulthood [302]. Moreover, BMAA modifies neuroblast organization increasing the number of neuroblasts clusters [303]. 

Recent studies have revealed that repeated rat treatment with MC-LR had a toxic effect on the development of a nervous system in the rat offspring [304]. 

### 4.2. Blood-Brain Barrier (BBB)

The BBB and the blood-cerebrospinal fluid (CSF) barrier separate the central nervous system (CNS) from blood and include the endothelial lining of the brain capillaries associated with astrocytes, pericytes, and neurons. The pericytes and astrocytes are closely associated with the endothelial cells and are required for capillary maturation (pericytes) and the maintenance of the permeability-barrier functions (astrocytes). The basement membrane (which contains laminin, proteoglycans, fibronectin, collagen IV, nidogen, and entactin) is essential for blood-barrier differentiation. BBB separates neurons from the circulating blood and maintains the internal chemical composition of the brain "milieu" responsible for the proper functioning of neuronal circuits, neurogenesis, angiogenesis, synaptic transmission, etc. BBB breakdown due to disruption of the tight junctions may result in synaptic and neuronal dysfunction and contribute to neurodegenerative disorders such as ALS, AD, Parkinson’s disease, and multiple sclerosis [305,306,307,308]. 

In adult rodent biological models, the rate of retention of BMAA is low [71,298,309,310]. Although Karlsson and co-authors [298,311] demonstrated a mild neuronal loss in the hippocampal regions of adult rats following BMAA exposure, no adult rodents models have successfully reproduced neuropathological changes typically seen in ND patients [312,313,314]. However, in rodent fetuses and neonates, early-life exposure to BMAA affects brain development with long-term consequences [33,298]. Moreover, neonatal exposure to BMAA during the critical period of neurogenesis caused β-amyloid deposition, neurofibrillary tangles of hyper-phosphorylated tau, Lewy bodies formation, microgliosis as well as neuronal loss in the hippocampal striatum, substantia nigra region and ventral horn of the spinal cord [315]. 

Berntzon and co-authors found BMAA in the CSF of a patient with ALS and some controls, though they did not confirm a prevalence of BMAA findings in the majority of ALS patients [121]. Significant amounts of BMAA were found in the brain tissues of ALS and AD patients, confirming the ability of BMAA to cross the BBB [122]. The other possible route of entry to the CNS is through the olfactory epithelium and the nasal passage or via the blood. The cyanobacterial neurotoxin BMAA can be directly transferred through olfactory pathways circumventing the BBB in mice and directly affecting olfactory neurons [260]. Recently, Garamszegi and co-authors [316] used triple quadrupole tandem mass spectrometry to demonstrate that BMAA and its isomers AEG and 2,4-DAB were detected in olfactory tissues of AD post-mortem brains. This finding contradicts early reports from Meneely and co-authors, who did not find BMAA in the brains of AD patients [124]. 

MC-LR has been confirmed to cause BBB disruption and enter the brain tissue, resulting in neurotoxicity and inducing structural and functional changes in neuronal cells [317,318,319]. Moreover, MC-LR had increased BBB permeability in mice inducing metalloproteinase-8 (MMP-8) expression and breaking through tight junctions [320]. MCs exposure inhibits serine- and threonine-specific protein phosphatases immune cortical neurons [321,322,323]. Recently, a study by Yan and co-authors [324] revealed that MC-LR disrupted the function of the neuronal ubiquitin-proteasome system in neurons, leading to the release of α-synuclein (α-syn) from neurons. The presence of α-syn in Lewy bodies has been associated with several ND [325,326]. In addition, α-syn was transported into glial cells through TLR4 receptors and activated the NLRP3 inflammasome [324].

### 4.3. Glia

Glial cells, including microglia, have long been suspected of playing a role in AD, but only because of their ability to react to neuronal dysfunctions (e.g., amyloid and Tau aggregates). Microglial activation and neuroinflammation are common to many NDs. This neurocentric view, which considered glial cells as secondary, has been challenged recently by the results of genetic association studies identifying genetic loci associated with the risk of AD that is associated with genes preferentially or exclusively expressed in glial cells [327]. 

The research on cyanopeptides effects on glia is limited. Chiu and co-authors [328,329] demonstrated a gliotoxicity of BMAA using the olfactory ensheathing cell as an in vitro model. A study conducted by Bubic and co-workers [330] showed that depsipeptide planktopeptin and anabaenopeptins impair the metabolic activities of normal human astrocytes via membrane perforation, oxidative stress, and changes in mitochondrial metabolism. Later, Mello and co-authors [331] showed cytotoxic effects of BMAA and MC-LR on primary astrocytes isolated from mixed adult brain cell cultures; and Soto, with co-workers [332], demonstrated damaging BMAA effects on Muller’s glial cells. Both glial cells and neurons can uptake and accumulate BMAA, as demonstrated using a specific polyclonal antibody against BMAA [333]. BMAA induces a proinflammatory response in astrocytes and microglial cells, causing a shift in the ratio of CD86/CD206 cells [303,334]. 

Takser and co-authors [335] demonstrated the effects of low doses of MC-LR, alone or as a part of a complex mixture with other cyanotoxins, such as cylindrospermopsin and anatoxin-A, on the viability of murine glial and neuroblastoma cell lines.

The role of dysfunctional astrocytes in the pathogenesis of ALS and other ND indicates that astrocytes may be targeted with strategies for their revival. These strategies may include direct intervention on astrocytes with modulatory medicines, exosomes, and miRNA-based therapies or their replacements. 

## 5. Cyanotoxins, Cyanopeptides and Neurodegenerative Diseases

A central dogma of age-related ND is the claim that the accumulation and propagation of aggregated proteins cause neurodegeneration [336]. Recently, a mechanism that does not involve a specific neuropathogenic protein but is mediated by error-prone translation leading to stochastic near-cognate missense substitutions was suggested. Drummond and Wilke proposed in 2008 that tolerance to translation errors of certain proteins provides a new mechanism to explain their propensity to misfold pathologically [337]. Mistranslation destabilizes the proteome leading to misfolding and accumulation in the cells of potentially toxic protein aggregates [67,337]. The finding that translational error increases with age in some biological models (*Drosophila*) [338] may suggest the possibility that the rate of translation leading to aging-related proteostasis failure may be a key event in early ND diseases [339]. The mistranslating cells exhibit severely inhibited protein synthesis and formation of protein aggregates in the cellular ND model [340]. Aminoacyl-tRNA synthetases (AARSs) catalyze covalent binding tRNA with their cognate amino acids and are two-to-three orders of magnitude more selective than other amino acid-utilizing [341]. Altered translation fidelity impairs cellular homeostasis and has been implicated as a molecular mechanism underlying changes in synaptic function, selective loss of certain types of neurons, and the pathogenesis of numerous ND [342,343,344].

Hundreds of non-proteinogenic amino acids produced by cyanobacteria include BMAA and can, in principle, enter human protein synthesis through foods and drinking water. Many non-proteinogenic amino acids have structures similar to the standard amino acids and have the potential to be misincorporated into the proteins [345]. It has been speculated that microbial-produced non canonical amino acids could represent one of the environmental triggers for the onset of neurodegeneration [62,346]. 

**Figure 1:**
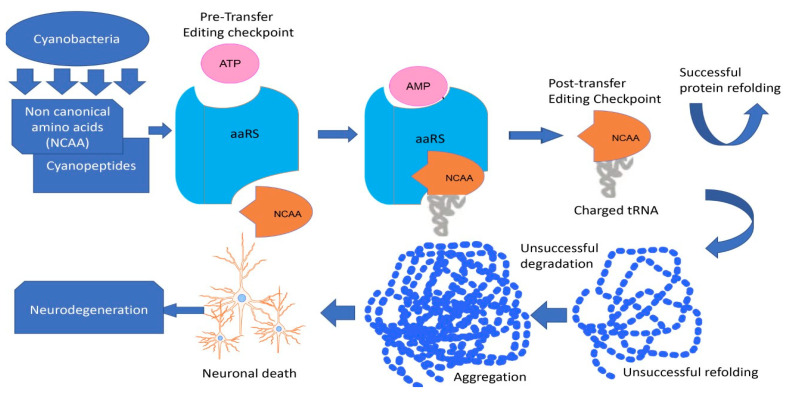
Fate of non-canonical amino acids (NCAA) produced by cyanobacteria in the neuronal cell. Misfolded proteins can be refolded via chaperones or degraded; forming of aggregates can lead to neuronal cell death.

The BMAA provides one of examples of multiple mimicry [345]. Earlier studies support the ability of BMAA to be incorporated into the proteins [57,347]. It has been hypothesized that BMAA is misincorporated at serine codons during protein synthesis [57]. The detection of non-canonical amino acids in protein sequence employed evaluation of misincorporation at the tRNA level using radiolabelled amino acids and tRNA microarrays to detect misacylation. This approach was used to prove that BMAA charges to both alanine and serine tRNAs and bypasses the proofreading ability of the alanine aaRs, suggesting misincorporation at alanine positions [72,188]. Han and co-authors [72] demonstrated that BMAA is a substrate for human alanyl-tRNA synthetase (AlaRS) and not a substrate for human seryl-tRNA synthetase (SerRS), and can form BMAA-tRNA-Ala by escaping from the intrinsic AlaRS proofreading activity (Figure 1).

Furthermore, BMAA inhibits the cognate amino acid activation, the editing functions of AlaRS, and the deacylation activity of HsAlaRS on Ser-tRNAAla [72]. The AlaRS possesses canonical and non-canonical cellular functions and is predominantly linked to loss in neuronal cells and neurodegenerative disorders in human and mouse models [67,343]. Moreover, using transcriptomic analysis, Wang and co-authors [348] confirmed that BMAA could alter the expression of major genes encoding components related to translation in prokaryotes (diazotrophic algae *Anabaena*). 

**Figure 2:**
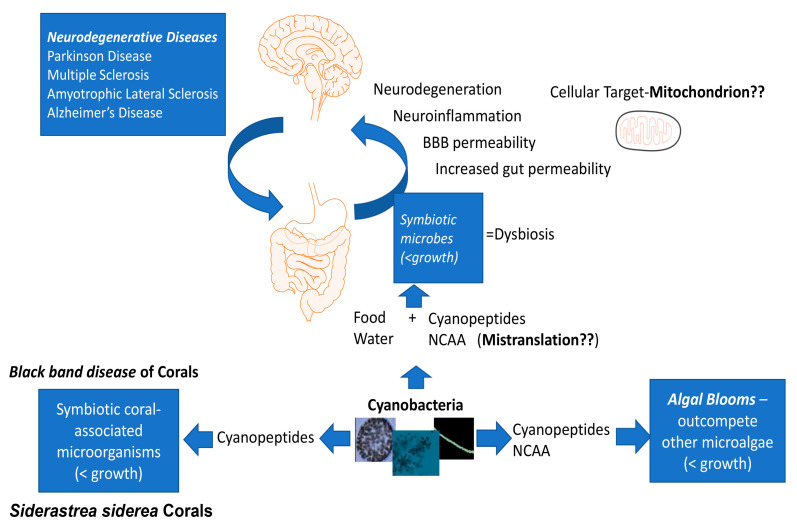
“Cyanopeptides Hypothesis”. NCAA and cyanopeptides production by cyanobacteria that leads to mistranslation, proteostasis, and cellular growth delay may be an ancient mechanism developed initially to regulate the growth of other microorganisms and outcompete them. Outcompeting of symbiotic microorganisms may lead to disease development (examples: (1) Black Band disease of corals associated with cyclic peptides production by *Roseophilum* sp. cyanobacteria [350]; (2) association of the gut dysbiosis and ND such as Parkinson’s and Alzheimer’s diseases [351,352]). The mitochondria (likely evolved from prokaryotic endosymbionts) and cells that are high-energy demanding, such as neurons and cardiomyocytes, are likely to be affected by NCAA (example: BMAA induce mitochondrial dysfunction in neurons with cardiolipin exposure [353,354] and in embryonic cardiomyocytes leading to cardiac developmental defects [355].

We hypothesize that the ability of BMAA and other NCAA and cyanopeptides to affect protein homeostasis may have ancient evolutionary origins, initially serving to hinder the growth of neighboring microalgae in plankton communities. The inhibition of cell growth and progression in the cell cycle of eukaryotic cells was demonstrated in in vitro experiments [349]. The production of cyclic peptides, including non-proteinogenic amino acids, leads to the lysis of cyanobacteria and may be an effective control mechanism of cyanobacterial density during algal blooms [137]. A Trojan Horse entry of non-proteinogenic amino acids from cyanobacteria to eukaryotic proteins may be a more general event and not only limited by BMAA and may be reflected in associations with ND diseases (Figure 2).

## 6. Conclusions

The structural variety of cyanotoxins and cyanopeptides is produced during cyanobacterial blooms. Many structural aspects of key metabolites involved in the cyanotoxins pathways have yet to be elucidated. However, it is becoming clear that non-proteinogenic amino acids, free-existing or initially a part of cyanopeptides, may affect protein homeostasis and lead to mistranslation and misfolding of proteins in eukaryotic cells, building a link to ND development, including Parkinson’s and Alzheimer’s diseases. The gut symbiotic microorganisms become affected, and the development of chronic dysbiosis may increase gut and BBB permeability. Many aspects pertaining to the regulation, role, and function of cyanobacterial compounds also requires the development of innovative detection approaches. This knowledge may be harnessed to identify novel biomarkers for ND and new targets for interventions. 

## Figures and Tables

**Table 1:** ALS/PD clusters related to environmental factors and cyanobacteria.

Location	Period	ALS/PD Cases	Water Quality	Toxic Food or Dietary Components	Key Findings	Reference
Guam	1945–1969	492	Mn↑	Cycad flour	Cycad toxic effect; Biochemical and neuropathologic abnormalities in ALS/PD diagnosed locals	[98]
Guam	1940s–1960s	-	-	Cycad flour, flying foxes; food containing phytotoxins	Accumulation of cycad neurotoxins (BMAA, cycasin) in flying foxes; Flying foxes consumption → ALS-PDC	[99]
Guam and other Mariana Islands	1956–1980	39	-	-	Similar genotypic composition of Chamorros on all the Mariana Islands but different mortality rates of ALS/PD on Saipan than on Guam; Environmental factors of ALS > genetic	[100]
Guam, Canada	-	23	HABs: BMAA	Cycad flour, flying foxes (for Guam)	BMAA in tissues from frontal cortex; BMAA-containing food relates to ALS/PDC; HABs → cyanobacterial contamination water supplies →BMAA biomagnification	[101,102]
Kii Peninsula, Japan	1961	>4	Ca, Mg, Na, KHCO3, Cl ↓	-	Low mineral content in water supplies possibly leads to ND; > ALS—Mitogawa area	[103]
KII Peninsula, Japan	1972	40	Mn↑	-	Possible association of Mn to ALS.	[104]
Skaraborg, Sweden	1973–1984	70 males	-	-	Cluster of MND in Skaraborg; Agricultural occupation → MND risk.	[105]
Two Rivers, Small Wisconsin, USA	1975–1983	6	-	fish	Polychlorinated biphenyl Contaminated fish consumption → ALS risk.	[106]
France	1975–1999	18	-	-	ALS cluster in south-eastern France; Infections or environmental factors of ALS > genetic.	[107]
Italy	1980–2001	634	-	-	16 ALS clusters; Low efficiency in detoxification systems; Environmental factors of ALS (toxins)	[108]
Finland	1985–1995	576	Pb, Cd, Zn↑	-	Two ALS clusters; Environmental factors of ALS.	[109]
Enfield, NH, northeastern USA	1990–2007	278	HABs: BMAA, MC	Fish, shellfish	High ALS incidence near Lake Mascoma; Chronic exposure to cyanotoxins → ALS; Combined impact of multiple cyanotoxins.	[95]
Iraq, Saudi Arabia	1991–2001	48	BMAA	-	48 ALS cases in Persian Gulf war veterans linked to desert’s crust contains BMAA; Aerosolization of cyanobacteria → inhalation of dust → BMAA exposure	[110,111,112]
Southern France, Hérault district	1994–2009	381	HABs: BMAA	shellfish	ALS cluster in Thau lagoon; Association with high concentrations of BMAA in mussels and oysters	[113]
Northern New England, USA	1997–2009	688	HABs, [CH3Hg]+	-	11 clusters of ALS grouped in 4 regions; Location of ALS cases are close to water bodies where HABs occurs; Environmental factors → ALS risk	[114]
Northern New England, USA	1997–2009	>800	HABs	-	HABs → water-quality → ALS risk	[115]
Northern New England, USA	1999–2009	-	HABs: BMAA	-	Mapping cyanobacterial HABs for northern New England lakes; Cyanotoxins increase ALS risk.	[97]
Western NH, USA	-	-	HABs: BMAA	fish	High concentrations of BMAA and DAB were found in the Lake Mascoma fish; BMAA, DAB, AEG in the air filters; ALS linked to BMAA.	[96]
France	2003–2011	72	HABs: BMAA		Nine ALS clusters; ALS linked to BMAA.	[116]
South Korea	2005–2017	-	HABs: BMAA mycrocystin, and other cyanotoxins	-	HABs severity → ND occurrence; HABs→ long-term impacts on human health	[117]
Guadeloupe	1996–2011	63	-	-	The highest incidence of ALS - Marie-Galante island; Environmental factor(s) → ALS risk	[118]
Northern and Southern Italy	2002–2012	95	-	dietary supplements	Private wells using → ALS risk↑; Amino acid supplements → ALS risk	[119]
Annapolis, Maryland, USA	2013	3	HABs: BMAA	blue crab	High concentrations of BMAA in the crabs originated Chesapeake Bay HABs exposure → ALS occurrence	[120]

**Table 2:** Chemical detection methods for cyanotoxins in water samples.

№	Cyanotoxins	Detection Techniques	Sensivity		Reference
			LOD	LOQ	
1.	MC-LR and 2 congeners	UHPLC-MS/MS	0.02–0.04 ng/mL	-	[185]
2.	MC-LR and 11 congeners	UHPLC-MS/MS	-	0.2 µg/L	[186]
3.	MC-LR and 4 congeners	LC-MS/MS	0.005–0.0817 µg/L	0.005–0.0817 µg/L	[187]
	Nodularin		0.0048 µg/L	0.0048 µg/L	
	Anatoxin-a		0.0001 µg/L	0.0004 µg/L	
	Cylindrospermopsin		0.0001 µg/L	0.0004 µg/L	
4.	BMAA	UHPLC-MS/MS	0.02 pg/µL	0.05 pg/µL	[188]
	2,4-DAB		0.04 pg/µL	0.13 pg/µL	
5.	MC-LR and 7 congeners	LC-MS/MS	-	0.04–0.5 µg/L	[189]
	Anatoxin-a		-	0.02 µg/L	
	Cylindrospermopsin (and deoxyCYN)		-	0.01–0.02 µg/L	
	Saxitoxins (4 congeners), GTX (5 congeners), decarbamoylgonyautoxin, N-sulfogonyautoxins-1 and -2		-	0.1–2 µg/L	
6.	MC-LR and 11 congeners	HPLC-MS/MS	0.01 ± 0.01–0.19 ± 0.2 μg/L	0.04 ± 0.04–0.64 ± 0.65 μg/L	[190]
	Nodularin		0.04 ± 0.02 μg/L	0.13 ± 0.06 μg/L	
7.	MC-LR and 2 congeners	HPLC-UV/PDA	3–4 μg/L	9–13 μg/L	[191]
8.	MC-LR and 2 congeners	HPLC-HRMS	0.002 μg/L	-	[192]
9.	Anatoxin-a,	HILIC-MS/MS	0.004 ng/mL	0.01 ng/mL	[193]
	Cylindrospermopsin		0.07 ng/mL	0.23 ng/mL	
	Saxitoxin		0.01 ng/mL	0.04 ng/mL	
	MC-LR and 4 congeners	RPLC- MS/MS	0.02–0.08 ng/mL	0.07–0.28 ng/mL	
	Nodularin		0.05 ng/mL	0.18 ng/mL	
10.	MC-LR and 5 congeners	UHPLC-MS/MS	-	0.046–0.052 µg/L	[194]
	Nodularin		-	0.049 µg/L	
	Cylindrospermopsin		-	0.052 µg/L	
11.	BMAA	UHPLC-HRMS	5 ng/L	10 ng/L	[195]
	DAB		3 ng/L	5 ng/L	
	AEG		2 ng/L	5 ng/L	
	BAMA		5 ng/L	10 ng/L	
12.	MC-LR and 5 congeners	HPLC-MS/MS	0.0003–0.0009 µg/L	-	[196]
	Cylindrospermopsin		0.0005 µg/L	-	
	Saxitoxin, dcSTX		0.0009–0.0013 µg/L	-	
13.	BMAA	LC-MS/MS	10 ng/L	-	[197]
14.	MC-LR and 5 congeners	LC-MS/MS	0.04–0.8 μg/L	0.1–2.3 μg/L	[198]
	Nodularin		0.3 μg/L	0.9 μg/L	
	Anatoxin-a		0.27 μg/L	0.81 μg/L	
	Cylindrospermopsin		0.14 μg/L	0.4 μg/L	
15.	MC-LR and 2 congeners	HPLC-DAD	0.08–0.15 µg/L	-	[199]
16.	MC-LR and 11 congeners	LC-MS/MS	0.001–0.007 μg/L	0.003–0.020 μg/L	[200]
	Nodularin		0.002 μg/L	0.006 μg/L	
	Anatoxin-a		0.001 μg/L	0.003 μg/L	
	Cylindrospermopsin		0.001 μg/L	0.003 μg/L	
17.	MCs	UPLC-MS/MS	0.005 µg/L	-	[201]
	Anatoxin-a		0.02 µg/L	-	
	Cylindrospermopsin		0.02 µg/L	-	
	Saxitoxin		0.8 µg/L	-	
	BMAA		0.03 µg/L	-	
18.	BMAA	LC-MS/MS	0.030 μg/L	0.096 μg/L	[202]
19.	MC-LR and 11 congeners	on-line SPE – UHPLC-HRMS	5–37 ng/L	15–130 ng/L	[203]
	Anatoxin-a		15–18 ng/L	50–60 ng/L	
	Homoanatoxin-a		11–12 ng/L	30–40 ng/L	
	Cylindrospermopsin		41–53 ng/L	130–170 ng/L	
20.	MC-LR and 1 congener	HPLC-DAD	0.2-0.3 μg/L	-	[204]
21.	Anatoxin-a	UHPLC-MS/MS	1.1 ng/L	2.5 ng/L	[205]
	Cylindrospermopsin		10.9 ng/L	21.7 ng/L	
	Saxitoxins (4 congeners)		3.5-9 ng/L	7.1–26.9 ng/L	
	GTX (7 congeners)		18.5–54.5 ng/L	42.2–227.6 ng/L	
22.	MC-LR and 7 congeners	UHPLC-MS/MS	0.1 µg/L	0.5 µg/L	[181]
	Nodularin		0.1 µg/L	0.5 µg/L	
23.	MC-LR and 2 congeners	UHPLC-MS/MS	0.1 µg/L	24 µg/L	[206]
24.	Cylindrospermopsin	UHPLC-MS/MS	0.029 μg/L	0.091 μg/L	[207]
25.	Saxitoxins (4 congeners)	on-line SPE–HILIC-HRMS	0.72–3.9 ng/L	2.4–13 ng/L	[208]
26.	MC-LR and 1 congener	tandem-SPE-HILIC-MS/MS	0.0012–0.0021 μg/L	0.004–0.007 μg/L	[209]
	Nodularin		0.0021 μg/L	0.007 μg/L	
	Anatoxin-a		0.03 μg/L	0.1 μg/L	
	Cylindrospermopsin		0.0012 μg/L	0.004 μg/L	
	BMAA		0.015 μg/L	0.05 μg/L	
	DAB		0.009 μg/L	0.03 μg/L	
	AEG		0.006 μg/L	0.02 μg/L	
27.	BMAA	LC-MS/MS	2.8 ng/mL	-	[210]
	DAB		1.7 ng/mL	-	
28.	BMAA	on-line SPE-UHPLC-HRMS	10 ng/L	-	[211]
	BAMA		10 ng/L	-	
	DAB		10 ng/L	-	
	AEG		5 ng/L	-	
29.	BMAA	UHPLC-MS/MS	-	2.5 µg/L	[212]
	AEG		-	2.5 µg/L	
	DABA		-	5 µg/L	
30.	MC-LR and 7 congeners	UHPLC-MS/MS (ESI)	0.02–0.2 µg/L	0.05–0.5 µg/L	[213]

LOD—limit of detection; LOQ—limit of quantification; UHPLC—ultra high-performance liquid chromatography; HILIC-MS/MS—hydrophilic interaction liquid chromatography-tandem mass spectrometry; RPLC-MS/MS—reverse phase chromatography tandem mass spectrometry; UV/PDA—ultraviolet/photodiode array detection; DAD—diode array detector; ESI—electrospray ionization; SPE—solid phase extraction; BAMA—β-amino-N-methylalanine; GTX—gonyautoxins; dcSTX—decarbamoylsaxitoxin.

## Data Availability

The data presented in this study are available in this article.
